# The role of pharmacogenomics in personalized medicine

**DOI:** 10.3325/cmj.2025.66.379

**Published:** 2025-12

**Authors:** Nada Božina, Julia Carolin Stingl

**Affiliations:** 1 Department of Pharmacology, University of Zagreb School of Medicine, Zagreb, Croatia (retired); 2 Department of Clinical Pharmacology and Pharmacoepidemiology, Heidelberg University, University Hospital Heidelberg, Heidelberg, Germany

In this century of personalized medicine, the standard we strive for is treatment based on an individualized approach. Applying an integral holistic approach to solving complex multifactorial drug-related problems could optimize pharmacotherapy. Pharmacogenomics (PGx) studies address how gene (pharmacogene) variants carried by individuals affect their response to drugs. These data can be used to select the drug and dose, increasing the likelihood that each person will receive the optimal dose of the most effective drug for them on their first treatment.

According to the Clinical Pharmacogenomics Database (ClinPGx, www.clinpgx.org), over 90% of the general population, of different ethnicities, carries at least one high-risk pharmacogenetic variant that modulates drug-body interactions. Pharmacogenetic testing has been confirmed as a promising clinical tool for the optimization of medication management, significantly improving drug safety and clinical outcomes that contribute to the overall health care cost reduction (1-3). PGx approach is particularly valuable for individuals with multimorbidity and polypharmacy, who also have higher hospitalization rates and mortality.

There is evidence that 5%-15% of hospitalizations in adults and 15% among patients with multimorbidities are due to adverse drug reactions (ADRs), with a negative impact on clinical outcomes and a burden on health care resources (4-6). Furthermore, more than 80% of ADRs associated with hospital admissions or occurring during hospitalization are dose-dependent, which suggests they could have been prevented (7).

The potential consequences of pharmacogenetic variability in drug metabolism and transport are an increased risk of side effects and therapeutic failure. The first studies on genotype-based drug dose adjustment were published 20 years ago. The focus was on drugs that are substrates of polymorphic cytochrome P450 (CYP) enzymes, such as antipsychotics and antidepressants (8,9).

Considerable progress in PGx has been achieved through the establishment of professional societies such as Clinical Pharmacogenetics Implementation Consortium (10) and the Dutch Pharmacogenetics Working Group (11). These societies, by issuing guidelines and recommendations (genetic-based dose adjustments), or by suggesting alternative medications, create the basis for the translation of knowledge from bench to bedside, facilitating the adoption by clinicians and application in practice. Guidelines are the result of a systematic review of relevant evidence-based literature on the associations of a drug with specific gene variants, and expert consensus on the strength of the published evidence (12).

Pharmacogenetically guided pharmacotherapy has been proven to be a useful tool for minimizing the risk of ADRs. A particularly promising approach is to use comprehensive panels for preventive genotyping instead of reactive testing of a single gene for drug effects. The feasibility of using different genetic panels in clinical practice has been tested in several implementation studies. In the Preemptive Pharmacogenomic Testing for Preventing Adverse Drug Reactions (PREPARE) study, pharmacogenetic-guided therapy, including a 12-gene pharmacogenetic testing panel, significantly reduced (by 30%) the incidence of clinically relevant ADRs in different disease areas and European health care systems (13).

Although significant progress has been made in the field of PGx, translation into real-world practice has not kept pace (14). The obstacles to the adoption of PGx achievements in the clinic are reimbursement issues, cost-effectiveness, awareness and education of health professionals, limited exchange of medical/laboratory records, and disagreements on the required level of evidence, as well as technical challenges.

Besides, PGx currently does not investigate the role of rare gene variants, and the transferability of pharmacogenetic guidelines for patients of different races and ethnicities is questionable, which should also be addressed in future studies. One of the important unanswered questions is the role of PGx in polypharmacy.

Among the predisposing factors for the development of ADRs are drug-drug interactions (DDIs) and drug-gene interactions (DGIs), significantly contributing to hospitalizations and in-hospital mortality. Pharmacokinetic (PK) DDIs often occur at the level of cytochrome P450 (CYP) enzymes or ATP-binding cassette (ABC), drug transporters involved in the absorption, distribution, metabolism, and excretion (ADME) processes of many clinically used drugs.

Particularly important for the translation of genotypes into the corresponding phenotypes is the occurrence of phenoconversion. In cases of polypharmacy and inhibition of enzymes involved in the metabolism of concomitantly administered drugs, phenoconversion may result in a discrepancy in the prediction of phenotype according to genotype, which reduces the relevance of an individual pharmacogenetic profile. Phenoconversion models described in the literature can help in predicting the effects of drug interactions, taking into account the pharmacogenetic status of patients (15). In the real world, DDIs and DGIs often occur simultaneously in the form of drug-drug-gene interactions (DDGIs), making clinical decisions difficult. Conducting DDGI studies and extrapolating the obtained results into recommendations for clinical use is very complex. Concepts that use physiologically based pharmacokinetic modeling to investigate such demanding situations can help in handling patient-specific DDGIs at the bedside (16).

Since the ultimate goal is to make pharmacogenomic-based therapy available for all, implementation should be improved through educational programs, recommendations issued by regulatory authorities, and guidelines dealing with test harmonization and interpretation, which should all result in better acceptance by patients and health care professionals.

Advancing bioinformatic tools can enhance pharmacogenomic data analysis and interpretation, thereby improving the implementation of PGx guidelines for the clinical decision support system (Clinical Decision Support System).

Among the first PGx guidelines issued were those for the treatment of cardiovascular diseases, proving the significance and feasibility of this type of guidelines. But despite the availability of genetic tests for coumarin anticoagulants, clopidogrel, some statins, metoprolol, flecainide, and propafenone, pre-emptive testing is rarely adopted in daily clinical practice. For some other cardiovascular drugs, such as direct oral anticoagulants, there are still no usable guidelines. For this reason, a research team from Croatia developed a project examining the role of pharmacogenomics in the prediction of adverse effects of cardiovascular drugs, with emphasis on the polygenic approach in testing DDGI. More about this promising project, the first of this kind focused on a European population of Slavic descent, you can read in the current issue of the *Croatian Medical Journal* (17).
